# Determinants of prompt and adequate care among presumed malaria cases in a community in eastern Rwanda: a cross sectional study

**DOI:** 10.1186/s12936-016-1285-7

**Published:** 2016-04-21

**Authors:** Chantal Marie Ingabire, Fredrick Kateera, Emmanuel Hakizimana, Alexis Rulisa, Claude Muvunyi, Petra Mens, Constantianus J. M. Koenraadt, Leon Mutesa, Michele Van Vugt, Bart Van Den Borne, Jane Alaii

**Affiliations:** Medical Research Center, Rwanda Biomedical Center, Kigali, Rwanda; Department of Health Promotion, Maastricht University, Maastricht, The Netherlands; Academic Medical Center, Amsterdam, The Netherlands; Malaria and Other Parasitic Diseases Division, Rwanda Biomedical Center, Kigali, Rwanda; Laboratory of Entomology, Wageningen University, Wageningen, The Netherlands; Department of Cultural Anthropology and Development Studies, Radboud University Nijmegen, Nijmegen, The Netherlands; College of Medicine and Health Sciences, University of Rwanda, Kigali, Rwanda; Royal Tropical Institute/Koninklijk Instituut voor de Tropen, KIT Biomedical Research, Amsterdam, Netherlands; Context Factor Solutions, Nairobi, Kenya

**Keywords:** Malaria symptoms, Healthcare seeking, Community, Health insurance, Theory of planned behaviour, Rwanda

## Abstract

**Background:**

In order to understand factors influencing fever/malaria management practices among community-based individuals, the study evaluated psychosocial, socio-demographic and environmental determinants of prompt and adequate healthcare-seeking behaviours.

**Methods:**

A quantitative household (HH) survey was conducted from December 2014 to February 2015 in Ruhuha sector, Bugesera district in the Eastern province of Rwanda. HHs that reported having had at least one member who experienced a fever and/or malaria episode in the previous 3 months prior to the study were included in the analysis. Healthcare-seeking behaviours associated with the last episode of illness were analysed. Socio-demographic, health facility access, long-lasting insecticidal-treated nets (LLINs), data on malaria knowledge, data and theory of planned behaviour (TPB) related variables (attitudes, subjective norms, perceived behavioural control) with regard to fever/malaria healthcare seeking, were collected. The primary outcome was prompt and adequate care defined as: (1) seeking advice or treatment at a health facility (health centre or hospital) or from a community health worker (CHW); (2) advice or treatment seeking within same/next day of symptoms onset; (3) received a laboratory diagnosis; (4) received advice or treatment; and, (5) reported completing the prescribed dose of medication. Determinants of prompt and adequate care among presumed malaria cases were evaluated using a logistic regression analysis.

**Results:**

Overall, 302 (21 %) of the 1410 interviewed HHs reported at least one member as having experienced a fever or malaria within the 3 months prior to the survey. The number of HHs (where at least one member reported fever/malaria) that reported seeking advice or treatment at a health facility (health centre or hospital) or from a CHW was 249 (82.4 %). Of those who sought advice or treatment, 87.3 % had done so on same/next day of symptoms developing, 82.8 % received a laboratory diagnosis, and more than 90 % who received treatment reported completing the prescribed dosage. Prompt and adequate care was reported from 162 of the 302 HHs (53.6 %) that experienced fever or malaria for one or more HH members. Bivariate analyses showed that head of household (HoH)-related characteristics including reported knowledge of three or more malaria symptoms, having health insurance, being able to pay for medical services, use of LLINs the night before the survey, having a positive attitude, perceiving social support, as well as a high-perceived behavioural control with regard to healthcare seeking, were all significantly associated with prompt and adequate care. In the final logistic regression model, a high-perceived behavioural control (odds ratio (OR) 5.068, p = 0.042), having a health insurance (OR 2.410, p = 0.044) and having knowledge of malaria symptoms (OR 1.654, p = 0.049) significantly predicted prompt and adequate care.

**Conclusions:**

To promote prompt and adequate care seeking for malaria in the area, particular emphasis should be placed on community-focused strategies that promote early malaria symptom recognition, increased health insurance coverage and enhanced perceived behavioural control with regard to healthcare-seeking.

## Background

Malaria is one of the major public health problems globally and according to the World Health Organization (WHO), about 3.2 billion people are at risk [1, 2]. Many control efforts have been deployed to reverse this high burden and between 2005 and 2015 declines in malaria incidence of 37 %, malaria-related deaths by 67 % across all age groups, and by 65 % among children aged under 5 years have been reported [2]. Despite these achievements, the sub-Saharan African region bears a disproportionately high global malaria burden: about 90 % of malaria cases and ≥90 % of malaria deaths [2]. In view of this, the WHO global initiative to fight malaria recommends prompt diagnostic testing, effective treatment and tracking of malaria cases to complement available scaled-up tools, including universal coverage of long-lasting insecticidal-treated nets (LLINs) and indoor residual spraying (IRS) with insecticide and treatment of malaria cases with artemisinin combination therapy (ACT) in malaria-endemic countries [3]. Early diagnosis and prompt treatment (within 24 h of symptom onset) are therefore key factors in reducing disease duration, complications and preventing mortality [4–7]. However at community level in Rwanda, no study has described adherence to the recommended early diagnosis and prompt treatment and hence the need for an evaluation of individual healthcare-seeking behaviours and their determinants in a rural, malaria-endemic setting and improvement of malaria-related health [8, 9].

### Malaria in Rwanda

In line with the sixth millennium development goals (MDGs) to fight malaria, Rwanda achieved a malaria morbidity decline from 73.5 % in 2002 to 7 % in 2012 among children under five [10, 11]. Malaria dropped from being the number one killer of children under five in 2005 to number three in 2008 and subsequently number 11 in 2011 [12]. The country is aiming to achieve malaria pre-elimination levels by 2018. The gains in malaria reduction observed so far were associated with scaled up interventions, including treatment with ACT and deployment of vector control strategies of LLINs and IRS with special emphasis on health system strengthening from highest to local levels [10–12].

Since 2005, Rwanda scaled-up free distribution of LLINs among children under five during immunization campaigns as well as among pregnant women in antenatal care [12]. This was followed by free universal coverage with LLINs to households (HHs) in the context of universal coverage in 2010 [12]. Since 2010, IRS with insecticides has been used in the most malaria-endemic zones in the eastern and southern provinces of Rwanda [10]. Lastly, case management using appropriate and effective anti-malarial drugs, such as ACT, has been adopted and scaled-up at community level as a key strategic intervention for malaria control countrywide [12].

### Rwanda health care system

Health services in Rwanda are provided through government-supported, non-governmental and privately owned health facilities with a minimum defined services package [13, 14]. Government-funded health facilities are offered along a hierarchical structure of central (referral hospitals), intermediate (provincial health offices) and peripheral (districts hospitals, sector level health centres and village level posts) levels. Health centres serve a catchment area of an administrative sector (~20,000 people) and offer an integrated package of preventive, curative, promotional, and rehabilitation services [13].

To increase access to health care services, Rwanda adopted the WHO recommended integrated community case management (ICCM) through community health workers (CHWs) in 2009 [15]. At village level, CHWs provide promotional and preventive services, including diagnosis and treatment of malaria among children under five, in addition to their other responsibilities [15].

With the goal of universal health coverage and achieving the MDG targets, Rwanda established a system of subsidized, community-based health insurance (CBHI) that has been a success, with around 78 % of the Rwandan population enrolling in 2010 [16, 17]. Apart from the CBHI, commonly called *Mutuelles*, in which ~98 % of the total population are insured, other insurance schemes include *La Rwandaise d’ Assurance Maladie* (RAMA), military medical insurance (MMI) and private insurances with coverage rates of 3.7, 0.6 and 0.4 %, respectively [16]. CBHI has been rolled out since 1999, adapted in 2004 and scaled-up in 2006 [17]. CBHI’s funding has been covered primarily by annual premiums of members based on their socio-economic status, while additional supplementary funding is provided by the Government and its partners [17]. CBHI covers both disease prevention and health facility-based medical services and has been credited with increasing the use of medical care services while reducing household out-of-pocket spending (by ~22 %) of the total health care spending: a proportion high but close to that targeted by WHO (15–20 %) [18, 19].

### Theory of planned behaviour

The theory of planned behaviour (TPB) as articulated by Ajzen Icek has been used since 1985 to conceptually explain three constructs (attitude, subjective norms and perceived behaviour control) that predict intention towards the performance of human behaviours [20]. The TPB theory defines attitude as ‘a disposition to respond favourably or unfavourably to an object, behaviour, person, institution, or event’ [21]. Subjective norms are defined as ‘the social influence or the impact of others on people’s perceptions and behaviours’ while perceived behaviour control refers to ‘the subjective probability that a person is capable of executing a certain course of action’ [21]. This theory has been demonstrated to be closely linked with psychosocial and human behaviours and used to explore care-seeking behaviours for infectious diseases, such as HIV/AIDS, tuberculosis as well as other health-related problems, including nutrition and mental health [22–25]. However, few studies have reported the use of TPB in relation to malaria, and in particular with regard to healthcare seeking. Only one study has successfully explored using TPB, the impact of an education intervention on the use of malaria preventive measures [26].

### Healthcare seeking and rationale

Healthcare-seeking behaviour is defined as ‘the series of actions that patients adopt’ in relation to their health concerns, including but not limited to symptom recognition and formulation of a treatment plan [4]. Determinants of early or delayed malaria care seeking that have been reported previously to include knowledge gaps on malaria symptoms, socio-economic status (SES), proximity to a health facility, provider-patient relationship, drug and staff shortages, health insurance and negligence [6, 7, 27–31]. As stated above, TPB constructs of attitudes, subjective norms and perceived behavioural control have been shown to significantly influence understanding and explain psychosocial-related behaviours in health [21, 32].

Although many studies have highlighted interventions that aim to alter healthcare-seeking behaviours as appropriate for malaria control, there is a lack of empirical data describing relationships between TPB constructs and community-based presumed malaria in Rwanda and elsewhere in Africa [33–38]. Previously, close associations between malaria prevalence and factors including age, gender, fever history, and use of existing malaria preventive measures such as bed nets, have been shown, including in Rwanda [33–38]. However, no study has explored factors that are related to fever/malaria care seeking in a community setting in Rwanda to inform the country’s goal of universal health coverage and optimal community-based malaria case management. As part of a larger project, called malaria elimination project for Ruhuha (MEPR), aiming at achieving malaria pre-elimination levels using community-oriented approaches in the Eastern province of Rwanda, this study aimed at identifying predictors of prompt and adequate care seeking among community-based presumed malaria cases.

## Methods

### Study area

The MEPR project is being implemented in Ruhuha sector, Bugesera district, Eastern province of Rwanda. The study area has a population of estimated ~23,893 individuals living in 5098 HHs. Administratively, the area consists of 35 villages that are grouped into five cells. The area is a low-malaria-endemic setting with slide positivity rates of 23 % among symptomatic health facility cases and 5 % among community-based asymptomatic cases [35]. Since June 2014, the MEPR project has rolled out malaria-related prevention messages provided by local Community Malaria Action Teams (CMATs) in the Ruhuha community: this presumably contributed to the increase in community knowledge.

### Study design and sample size

Data for this sub-analysis was drawn from a larger cross-sectional survey. Considering a sampling frame of 4522 sector HHs generated from an enumeration survey done prior IRS in the sector, a final sample of 1410 HHs was randomly selected [39]. Among the 1410 HHs visited, 45 HHs were omitted due to various reasons (refusal to provide informed consent, mobility outside the sector or no eligible person at HH for the time of interview) and were replaced by neighbouring HHs [40]. This survey was conducted between December 2014 and February 2015 and respondents were heads of households (HoHs) or their spouses. For this analysis, 302 HHs with at least one family member reporting having experienced a febrile and/or presumed malaria episode in the last 3 months before the study were included and only data on the last fever/malaria episode per HH were considered.

### Data collection

Briefly, the team adapted a structured questionnaire based on the demographic health survey (DHS) and malaria indicator survey [12, 16]. The questionnaire was designed and formatted into open data KIT (ODK) collect set-up using English language and installed on Samsung Galaxy 2 tablets [39]. Training and pre-testing sessions were conducted prior to the survey using locally translated (Kinyarwanda) paper forms. A team of ten field staff collected data on demographics (age, sex, occupation, education, residence), SES (occupation, ownership of house, land or animals), health insurance ownership, LLINs use, knowledge of malaria, fever management practices (initial choice of care, time to seeking advice or treatment after symptom onset, diagnosis testing before treatment, and completion of prescribed dose of medication). Scale questions were assessed based on a three point Likert scale–this was found the most appropriate during the pre-testing phase of questionnaire development. Field staff had undergone 10 days of theoretical training focusing on study objectives, content of the questionnaire and electronic data capture followed by practical sessions [40].

A written informed consent was solicited from HoHs or spouse prior to initiation of study procedures and participants were not provided with any form of reimbursement.

### Measurements

#### Dependent variable

Prompt and adequate care in the context of this analysis refers to early diagnosis of recent presumed malaria cases at HH level (within same/next day), treatment of the confirmed cases with effective medicine as well as adherence to medications prescribed. To generate a composite outcome index for prompt and adequate care among HHs’ recent fever/malaria cases at HH, five initiated actions were therefore combined. These included: (1) sought advice or treatment within same/next day after fever started; (2) sought advice or treatment either at the health centre/hospital or from CHWs. Seeking advice or treatment at local drugstores was not included as no diagnostic tests are performed in those entities; (3) had a diagnostic test performed; (4) fever/malaria treatment was prescribed; and, (5) patient completed prescribed medications. The prompt and adequate care variable was subsequently dichotomized into ‘prompt = 1’ and ‘non-prompt = 0’ care.

#### Independent variables

TPB constructs (attitudes, subjective norms and perceived behavioural control) sub-items were developed based on malaria diagnosis and treatment responses. Sub-items were all graded using a three-point Likert scale (not important, important, very important). Positive answers for attitudes, subjective norms and perceived behavioural control were considered for those with responses of ‘very important’ per construct item. Therefore, a dichotomous variable for each construct was created compiling all positive answers under a final positive result and all the other responses as a negative response. The dichotomous variable was ‘Yes’ (responded very important to all sub-items) or ‘No’ (did not respond very important to all sub-items).

Attitude was measured by asking three questions on the beliefs that: (1) seeking advice or treatment within 24 h is important; (2) testing before treatment is important; and, (3) malaria medicines cure. Three questions constituted the scale on subjective norms, including (1) spouse/family support in seeking advice or treatment within 24 h of symptoms onset; (2) spouse/family support receiving a diagnosis before treatment; and, (3) spouse/family support taking medicine as prescribed when the participant or a member of the HH became sick.

Two items were identified to measure the level of perceived behavioural control including (1) feels confident to decide to go to the health centre once malaria symptoms were present; and, (2) the ability to take anti-malarial medication when diagnosed with malaria.

#### Comprehensive knowledge on malaria cause, symptoms, and prevention

To determine the correct knowledge of malaria transmission, options including but not limited to female mosquito *Anopheles,* malaria mosquito bite, cold drinks, drinking/eating dirty foods, rain, and sugar cane were considered. Correct knowledge was attributed to the participants who only reported that either the female mosquito *Anopheles* or malaria mosquito bites were the transmitter of the disease while not reporting any known misconceptions mentioned above.

Having ‘knowledge of malaria symptoms’ was based on reporting following symptoms: having a fever, joint pain, headache, sweating and shivering. Reporting three and above symptoms was considered adequate knowledge of symptoms and was used as a cut-off. In the final step, two categories for knowledge of malaria symptoms were created by grouping participants that listed less than three malaria symptoms (0) and those that listed three or more malaria symptoms (1).

‘Malaria prevention knowledge’ was constructed as per the standard Rwandan national malaria control strategy: those listing either LLINs and/or IRS and not mixing with other less efficacious methods were categorized as having correct knowledge on malaria prevention (1), while the remaining participants were categorized as not having enough knowledge on malaria prevention (0).

### Ability to pay for medical services

Two items, namely ability to pay for medical services and medicines prescribed, were evaluated using a 3-level Likert scale (unable to pay at all, able to pay half, able to easily pay). Participants were considered as able to pay if they responded “able to easily pay” for both items. Accordingly, a composite categorical variable was created using ‘able’ (1) or ‘not able’ (0) to pay for medical services.

### LLIN use, health insurance and difficulties in accessing medical services

Three additional dichotomous variables were also included in the analyses. These were whether or not participants had used LLINs the night before the survey, had a health insurance and whether or not participants experienced difficulties in accessing medical services (Yes/No). Reported difficulties were further identified using the following items: proximity of the health facility, lack of transportation means, time spent at the health facility, health insurance ownership, affordability of medical services, commodity/drugs stock outs, lack of enough health care workers.

### Data analysis

Data were analysed using SPSS version 21.0 (IBM Corp, Armonk, NY, USA). Descriptive statistics using frequencies, mean and standard deviations were applied to summarize the data. For internal reliability checks of the TPB scales (attitude, subjective norms and perceived behavioural control), Cronbach’s alpha (α) was calculated based on the variance within scale items and the covariance of the items. A score of or above 0.70 was considered as adequate reliability [41]. To assess associations between potential independent predictors reported by other studies to predict malaria care seeking (demographics, SES, malaria management related knowledge (cause, symptoms, prevention), LLIN use, ability to pay for medical services, ownership of health insurance, reported difficulties in accessing medical services, and TPB constructs) and the outcome variable-prompt and adequate care, bivariate logistic regression analyses were performed. All significant variables at a *p* value level of ≤0.25 (knowledge of malaria symptoms, LLIN use, ability to pay for medical services, ownership of health insurance, attitude, subjective norms and perceived behavioural control) were further included in a multivariate logistic regression analysis using a backward (likelihood ratio test) stepwise model. A separate multivariate logistic regression analysis was conducted in which only the three TPB variables were included as the independent variables.

### Ethical considerations

The study obtained approval from the Rwanda National Ethics Committee (No. 20/RNEC/2014). Study participants were taken through an informed consent process before signing.

## Results

### Socio-demographic characteristics

Of the 1410 HH visited, 302 (21.4 %) had at least one person with fever in previous 3 months. The majority of participants who were interviewed were male HoHs (77.5 %) and the study participant median for age was 40.5 (± SD13.0) with an age range of 21–86 years. The median number of household members was five with a range of 1 to 13. Details are shown in Table 1.

**Table 1:** Distribution of head of household baseline characteristics

Variable name	Variable groups	Frequency—n (%)
Sex of HoH	Male	234 (77.5 %)
	Female	68 (22.5 %)
Age of HoH	Median (± SD)	43.3 (± 13.0)
HH member size	Median (± SD)	5.0 (± 1.9)
HH with at least one/more children under five	Yes	243 (80.4 %)
HH with at least one/more children under five with fever/malaria in the last 3 months	Yes	3 (1 %)
Family owns the house lived in	Yes	255 (84.4)
Family owns a piece of land	Yes	209 (69.2 %)
HoH main occupation	Farmer	276 (91.4 %)
	Public officer	4 (1.3 %)
	Self-employer	14 (4.6 %)
	Private officer	2 (0.7 %)
	Unemployed	4 (1.3 %)
	Other	2 (0.7 %)
HoH highest educational level	None	96 (31.7 %)
	Primary School	179 (59.3 %)
	Post primary/vocational	7 (2.3 %)
	Secondary or higher	20 (6.6 %)
HoH marital status	Never married	9 (3 %)
	Married	143 (47.4 %)
	Living together	78 (25.8 %)
	Separated/divorced	25 (8.3 %)
	Widowed	47 (15.6 %)
ITN ownership and use	No of HHs owning at least 1 bed net	274 (90.7 %)
	No of HH that used ITN the night before survey	224 (73.2 %)
HH sprayed in the last 6 months	Yes	274 (90.7 %)
HoH health insurance ownership	Yes	270 (89.4 %)
HoH ability to pay medical services and medicines	Yes	217 (71.9 %)

### Malaria control measures

The two major malaria preventive measures reported commonly were ownership and use of LLINs the night before the survey by 274 (90.7 %) and 224 (73.2 %), respectively, while in 274 (90.7 %) HH application of IRS with insecticides was reported in the 6 months before the survey.

### Malaria management related knowledge

Of the 302 participants, 242 (80.1 %) had correct knowledge about how malaria is transmitted, reporting that malaria infection was transmitted by a female *Anopheles* mosquito bite or mosquito bite. The majority of the participants (92.1 %) reported fever as the most common malaria symptom whilst 63.4 and 41 % reported headache and joint pain, respectively.

About 152 (50.3 %) reported either LLINs or IRS as common malaria preventive measures without mentioning any other less efficacious ways of prevention.

Almost all participants (99 %) mentioned the local health centre as the common place to seek care, while 19 (6.3 %), 18 (6 %) and 11 (3.6 %) cited hospitals, CHWs and drugstores, respectively.

### Healthcare-seeking practices

Among HHs with an individual reporting a fever/malaria episode, about 87.3 % reported seeking advice or treatment within same or next day of developing the symptoms while 3 and 5 % reported seeking care, >48 and <72 or more hours after fever onset, respectively. With regard to where advice or treatment was sought, 76, 3.6, 2.6, and 10.6 % reported the health centre, hospital, from the CHWs, or drugstores, respectively. A perception of fever/malaria severity was reported by more than half of participants (53 %) to be the commonest reason of advice or treatment seeking whereas mobilization by a CHW accounted for 15 participants (5 %). Overall, 250 (82.8 %) received a diagnostic test before treatment while medication was provided for 273 (90.4 %) with 278 (92.1 %) of those treated reporting to have completed their dose as prescribed.

A majority of 270 HoHs (89.4 %) reported having a health insurance while 219 (72.5 %) and 218 (72.2 %) reported being able to pay for both medical services and to pay for medicines, respectively.

Overall, 21 (7 %) reported not seeking care and about 32 % of the participants reported difficulties in accessing medical services. The reasons for difficulty in accessing healthcare included long time spent at the local health centre (14.6 %), lack of a health insurance (6.3 %), lack of enough healthcare providers (5.6 %), and being far from the health facility (3.7 %).

### Attitude, subjective norms, perceived behavioural control

Two-hundred and seventy-one (91.4 %) participants believed that it was important to seek advice or treatment within 24 h, to have a diagnostic test before treatment, and that anti-malarial medicines do actually cure (α = 0.71). With regards to subjective norms, 277 (91.7 %) reported being supported by family/partner in seeking advice or treatment within 24 h, testing before treatment as well as adhering to the treatment (α = 0.94). Being confident in taking a decision to go to seek care and taking anti-malarial medicines as prescribed if tested malaria positive (perceived behavioural control) was reported among 288 (95.4 %) participants (α = 0.79) (Table 2).

**Table 2:** Descriptive statistics for TPB constructs

Variable name	Variable groups	Frequency—n (%)
Attitude	Important to seek care in less than 24 h	284 (94)
	Important to have a diagnosis test before treatment	290 (96)
	Anti-malarial medicines do cure	290 (96)
	Favourable attitude score^a^	276 (91.4)
Subjective norms	Support by family/partner to seek care in less than 24 h	284 (94)
	Support by family/partner to get tested before treatment	284 (94)
	Support by family/partner to adhere to treatment	285 (94.4)
	Social support score^a^	277 (91.7)
Perceived behavioural control	Confident to take a decision to go to seek care	288 (95.4)
	Confident to take anti-malarial medicines as prescribed if tested malaria positive	295 (97.7)
	Perceived behavioural control score^a^	288 (95.4)

^a^Reflects the recoded Likert scale and includes participants who only responded ‘very important’ to each of the sub-scale questions

### Prompt and adequate care for presumed malaria

Prompt and adequate care: having sought advice or treatment on same/next day, had a diagnostic test before treatment, received treatment and completed the prescribed dose of medication was achieved in 162 out of 302 HH recent fever/malaria episodes (53.6 %) (Table 3).

**Table 3:** Descriptive statistics for prompt and adequate care

Variable name	Variable groups	Frequency—n (%)
Prompt and adequate care	Sought care the same/next day after fever started	263 (87.3)
	Sought care at the health centre/hospital or from CHWs	249 (82.4)
	Had a diagnostic test performed	250 (82.9)
	Fever/malaria experience was treated	273 (90.4)
	Patient completion of prescribed medications	278 (92.1)
	Composite prompt and adequate care score^a^	162 (53.6)

^a^Includes only participants who met all the five items of the composite prompt and adequate care score

### Bivariate logistic regression on prompt and adequate care for presumed malaria

By bivariate analysis, prompt and adequate care was not influenced by variables of HoH demographic (age, gender, marital status, education, occupation) and SES variables (ownership of land or house). Bivariate results are shown in Table 4.

**Table 4:** Univariate and multivariate determinants of prompt and adequate care

Variable	Variable group	Univariate OR (95 % CI), *p* value	Multivariate OR (95 % CI), *p* value
Sex	Female	1.303 (0.759–2.238), p = 0.337	
Age of HoH	–	0.992 (0.975–1.009), p = 0.363	
Marital status	Not married/divorced	1.548 (0.928–2.583), p = 0.094	
Education of HoH	–	1.104 (0.819–1.489), p = 0.517	
Family size	–	0.968 (0.860–1.089), p = 0.587	
Occupation	Non-farmer	0.586 (0.253–1.360), p = 0.213	
Own a house	No	1.250 (0.671–2.331), p = 0.482	
Own a piece of land	No	1.311 (0.714–2.408), p = 0.382	
Reported LLIN use	No	1.776 (1.062–2.969), p = *0.029*	1.455 (0.844–2.510), p = 0.178
Health insurance ownership	No	3.342 (1.491–7.492), p = *0.003*	2.410 (1.026–5.660), p = *0.044*
Ability to pay medical services	No	2.016 (1.211–3.355), p = *0.007*	1.623 (0.947–2.780), p = 0.078
Difficulties in accessing medical services	Yes	0.692 (0.425–1.127), p = 0.139	
Knowledge on malaria cause	Not reported either female Anopheles mosquito or mosquito bite	0.724 (0.408–1.287), p = 0.271	
Knowledge on malaria symptoms	Less than three symptoms	1.735 (1.069–2.817), p = *0.026*	1.654 (1.003–2.729), p = *0.049*
Knowledge on malaria prevention measures	Not reported IRS and LLINs	0.916 (0.508–1.652), p = 0.771	
TPB—attitude		2.350 (1.012–5.454), p = *0.047*	1.433 (0.551–3.727), p = 0.461
TPB—subjective norms		3.267 (1.322–8.073), p = *0.010*	1.691 (0.609–4.696), p = 0.313
TPB—perceived behavioural control		7.500 (1.649–34.117), p = *0.009*	5.068 (1.062–24.170), p = *0.042*

Italic values are significant at p < 0.05 level

Only use of LLINs (OR 1.776, p = 0.029), having knowledge of three or more malaria symptoms (OR 1.735, p = 0.026), having health insurance (OR 3.342, p = 0.003), being able to pay for medical services (OR 2.016, p = 0.007), and having a positive attitude (OR 2.350, p value = 0.047), being socially supported in regard to advice or treatment seeking (OR 3.267, p = 0.010), had a high perceived behavioural control (OR 7.500 p = 0. 009) towards taking a decision to seek prompt and adequate care and take their medication as prescribed showed a possible effect on seeking and receiving prompt and adequate care.

### Multivariate analysis

Using a backward stepwise logistic regression, the odds of participants having knowledge of three and more malaria symptoms were 1.6 times higher than those knowing less than three malaria symptoms with regard to promptly seek care (Table 4). Those with health insurance (OR 2.4) and those with high perceived behavioural control to actually decide to seek care and taking medicines as prescribed (OR 5.0) showed significant influence to promptly seek care.

Use of LLINs, positive attitude and subjective norms in regard to healthcare lack a significant effect while a borderline significant association was observed in regard to the ability to pay for medical services (Table 4).

A logistic regression analysis in which only the three TPB variables: attitude, subjective norms, and perceived behavioural control towards prompt and adequate care, were included showed that only perceived behavioural control was borderline significant (OR 4.616, p = 0.062).

## Discussion

The analysis explored factors related to community-based presumed malaria care seeking. Findings have shown that ownership of health insurance, having adequate knowledge of malaria symptoms and high-perceived behavioural control in regard to taking the decision to seek care significantly predicted prompt and adequate care. The primary choice of advice or treatment for malaria in the area was the local health centre. However, 11 % of the participants reported seeking advice or treatment in private drugstores implying treatment without a confirmed diagnosis.

The finding that knowledge of malaria symptoms is associated with prompt and adequate care is corroborated by research reported elsewhere [4, 29, 30, 42–44]. Prompt and adequate care was also found to be associated with ownership of health insurance in this analysis and this is in accordance with a study conducted previously in Rwanda and Ghana in which health insurance was reported as a strong determinant of healthcare demand as a result of a reduction in out-of-pocket spending [17, 27].

Contrary to other settings [6, 28, 30, 45], individual variables of age, gender, marital status, education, and occupation were not found to be associated significantly with prompt and adequate care in this analysis. This may possibly link to the fact that the large majority of the Rwandan population have a CBHI and through this scheme have high access to the available healthcare services as highlighted above [15–17].

Multiple studies have reported a close link between geographic proximity of the health facility and prompt and adequate care seeking [6, 27, 28, 46, 47]. However, this study did not find this relationship. This is probably due to the fact that 85 % of the Rwandan population live within one-and-a-half hours of a primary healthcare unit, thus considered as having access to healthcare [13]. Some other studies highlighted that proximity may not be enough to explain prompt and adequate care seeking alone, rather should be linked with other factors such as efficient healthcare providers and services, including the availability of medicines and provider attitudes and skills for patient counselling among others [31, 48]. However, the concept of bringing services close to the people remains important [49]. A recent study in the same area of Rwanda showed a need among community members to mandate and train CHWs to treat adults in addition to children under five as a means to reduce the time spent seeking services or waiting for services at health facility, leading to increased loss of work hours [33].

Based on key findings of this analysis, a number of recommendations can be suggested. Firstly, the perceived behavioural control of TPB, which was shown to be a significant predictor of prompt and adequate care in this analysis, needs to be enhanced, for example, through guided practices and skills training [50]. Future interventions might also include modelling best practices (through cinema shows, role plays and public lecturing) from successful stories and lessons learnt from less successful programmes to enhance the importance and intention of seeking advice or treatment within 24 h of symptom onset as well as to adhering to completing prescribed medications [51].

Secondly, effective and continuous educational sessions at community and health-centre level should put particular emphasis on symptom recognition to guide prompt and effective fever/malaria cases management.

Thirdly, there is a need for standardized procedures in regard to fever/malaria advice or treatment seeking in drugstores and it has been evident that private drugstores may contribute to the improvement of malaria case management elsewhere [52, 53]. Furthermore, active community participation in demanding better services during provider-patient interaction (i.e., demand for test before treatment) should be emphasized during community sensitization [51].

Fourthly, the importance of a CBHI in the Rwandan health system has been demonstrated in terms of increasing the use of health services [17]. Approaches aimed at improving health insurance coverage and sustaining the system should be further studied to enable a better operationalization at all levels of healthcare provision, in collaboration with local governance.

Findings should be considered in light of some limitations. Firstly, participants provided data related to the recent fever/malaria experiences in the 3 months before the survey. These data may be influenced by recall bias. Secondly, the study team interviewed HoHs or spouses to obtain data on presumed fever/malaria cases at the HH level. However, this is a standard way of collecting data related to HH members across many studies. Thirdly, the study may not have comprehensively captured the TPB variables with the used measurements. The distributions indicated that most participants scored favourably on these constructs. Fourthly, data presented is based on participants’ self reports, which may be associated with socially desirable answers. Lastly, the study setting has been exposed to malaria preventive messages by local community malaria action teams (CMATs), which contributed possibly to the high level of community awareness in regard to malaria compared to the rest of the country. Study findings may therefore not be generalizable to all settings in Rwanda.

Despite the limitations, an advantage of this community-based study provided real field findings rather than restricting data to a health facility setting that may not be population representative. Future studies may explore healthcare providers’ perspectives regarding appropriate health service delivery as complementary to the beneficiary perspectives on fever/malaria care seeking explored in the current analysis.

## Conclusions

The study demonstrated that about half of the participants sought prompt and adequate care at the health centre, or hospital or CHWs within same/next day of symptom onset, received prompt diagnosis and treatment and reported completing the prescribed medication dose. HoH malaria symptom recognition, perceived behavioural control and ownership of health insurance significantly contributed to prompt and adequate care. Strategies of strengthening educational sessions with regard to the highlighted factors and increasing the coverage of CBHI at community levels should be developed.

## Abbreviations

ACT: artemisinin-based combination therapy
CBHI: community based health insurance
CHWs: community health workers
CMATs: community malaria action teams
DHS: demographic health survey
HH: household
HoH: head of household
ICCM: integrated community case management
IRS: indoor residual spraying
LLIN: long lasting insecticidal nets
MDGs: millennium development goals
MEPR: malaria elimination program for Ruhuha
MMI: military medical insurance
ODK: open data kit
RAMA: la rwandaise d’ assurance maladie
SES: socio-economic status
TPB: theory of planned behaviour
WHO: World Health Organization
